# Combining Phase Advancement and Period Correction Explains Rushing during Joint Rhythmic Activities

**DOI:** 10.1038/s41598-019-45601-5

**Published:** 2019-06-27

**Authors:** Thomas Wolf, Cordula Vesper, Natalie Sebanz, Peter E. Keller, Günther Knoblich

**Affiliations:** 1 Department of Cognitive Science, Central European University, Október 6 utca 7, 1051 Budapest, Hungary; 2 Department of Linguistics, Cognitive Science and Semiotics, Aarhus University, Aarhus, Denmark; 3 Interacting Minds Centre, Aarhus University, Aarhus, Denmark; 4 MARCS Institute for Brain, Behaviour and Development, Western Sydney University, Penrith, NSW 2751 Australia

**Keywords:** Oscillators, Human behaviour

## Abstract

When people engage in rhythmic joint actions, from simple clapping games to elaborate joint music making, they tend to increase their tempo unconsciously. Despite the rich literature on rhythmic performance in humans, the mechanisms underlying *joint rushing* are still unknown. We propose that joint rushing arises from the concurrent activity of two separate mechanisms. The phase advance mechanism was first proposed in research on synchronously flashing fireflies and chorusing insects. When this mechanism is combined with a human-specific period correction mechanism, the shortened periods of individual intervals are translated into a tempo increase. In three experiments, we investigated whether joint rushing can be reliably observed in a joint synchronization-continuation drumming task. Furthermore, we asked whether perceptual similarities produced by the actions of different individuals modulate the joint rushing effect. The results showed that joint rushing is a robust phenomenon occurring in groups of different sizes. Joint rushing was more pronounced when the action effects produced by different individuals were perceptually similar, supporting the assumption that a phase advance mechanism contributed to rushing. Further control conditions ruled out the alternative hypothesis that rushing during rhythmic interactions can be explained by social facilitation or action mirroring effects.

## Introduction

When humans engage in synchronized, rhythmic joint activities, they tend to increase their pace unconsciously. Even though this phenomenon appears to be ubiquitous and well known among musicians, dancers and their audiences^1–5^, it has hardly been addressed in research on timing mechanisms in humans (except for two recent studies^6,7^). Thus, despite the rich literature on the cognitive and neural bases of rhythmic performance in humans^8,9^, the psychological mechanisms underlying *joint rushing* are still unknown.

We argue that if joint rushing indeed emerges from human interaction in contrast to purely individual processes, then predominant models of inter-subjective sensorimotor synchronization are incomplete. Specifically, they do not consider how sounds produced during synchronous, rhythmic joint actions are integrated to result in specific patterns of tempo drift. Thus, the systematic study of joint rushing has potential to lead to more adequate models of inter-personal coordination by identifying missing components. In this article, we first present evidence that joint rushing is a robust phenomenon that emerges in interpersonal sensorimotor synchronization. As an explanation for joint rushing, we then propose a combination of two mechanisms, a period correction mechanism, which is a standard component of models of human sensorimotor synchronization^10^, with a phase advance mechanism, a mechanism that has been proposed to regulate rhythmic synchronicity in some firefly species and chorusing insects^11^.

## Previous Research

Timing mechanisms in humans have been studied extensively with sensorimotor synchronization paradigms, yet tempo drift in time series has usually been considered to be a “methodological inconvenience”^12^ necessitating techniques for minimizing or eliminating it^8^. It is not surprising, then, that current models of interpersonal sensorimotor synchronization in humans do not account for tempo drift and can thus not explain why groups engaging in rhythmic joint activities tend to increase tempo over time. Two indications that this is a robust phenomenon come from recent studies^6,7^. These studies rule out several potential explanations for joint rushing. Okano *et al*. provided evidence against the hypothesis that joint rushing simply emerges from the faster tapper in a pair acting as a leader and thereby setting a faster tempo for the interaction^6^. Thomson *et al*. investigated the role of negative mean asynchrony^9^, i.e. the tendency to tap too early when synchronizing with an external timekeeper, without being aware of it. They concluded that the evidence speaks against negative mean asynchrony as the cause of joint rushing^7^.

A further explanation in musicians’ discussions of joint speeding is that increased arousal causes the tempo increase in group performance. Thus, joint rushing may be an instance of social facilitation^13^. Zajonc’s arousal-based theory of social facilitation^14^ states that the mere presence of a conspecific increases the level of arousal, and thereby facilitates dominant responses, leading to an increase in performance speed (i.e. audience effects). Such arousal-based theories of social facilitation appear to correspond well with the experience of musicians and dancers that joint rushing is more pronounced during an exciting performance than during rehearsal^1,3^. Another possible explanation comes from the literature on mirroring accounts, in which facilitating effects of similar actions are reported^15^. These effects could lead to joint rushing, when co-actors mirror each other’s actions.

### Present study

We propose that joint rushing arises from the concurrent activity of two separate mechanisms: (1) The phase advance mechanism, a mechanism that shortens single intervals and thereby brings about synchrony, and (2) a human-specific adaptive period correction mechanism that translates the shortened period of individual intervals into a tempo change by adjusting internal timekeepers.

**Figure 1:**
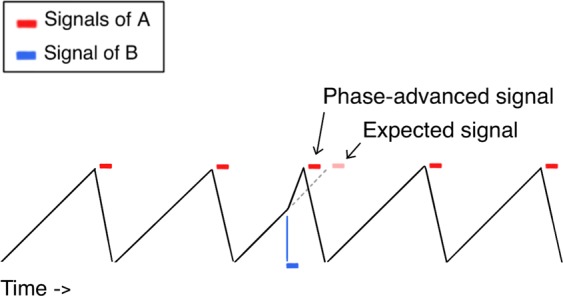
The phase advance mechanism is based on an oscillating timekeeper (indicated by the black sawtooth wave). Whenever the oscillating timekeeper reaches a threshold, signals (indicated by the red blocks) are triggered and broadcast after a short effector delay, and the oscillator is reset. If a conspecific’s signal (indicated by the blue block) falls into a certain temporal window before the threshold is reached, the current oscillation period is truncated and signaling is accelerated. The result is a phase advanced signal^11^.

The phase advance mechanism was first proposed in research on synchronously flashing fireflies and chorusing insects (crickets and cicadas) and also for certain anurans (some frogs)^16–20^. The mechanism assumes an oscillating timekeeper that initiates a signal whenever a signaling threshold is reached, followed by a resetting of the phase of the oscillator. The eponymous characteristic of this mechanism, however, is that it corrects phase differences between neighboring signalers by reducing the time it takes the lagging signaler to reach its signaling threshold (see Fig. 1), i.e., by advancing the phase of the lagging signaler. This advancement leads to a single shortened period. For the advancement to occur, a conspecific’s signal has to fall into a temporal, *sensitive window*. This window is partially determined by the fact that some time passes between the oscillator reaching its threshold and the signal being broadcast (see Fig. 1). Perfectly aligned signals would therefore never fall into the sensitive window and therefore never trigger the mechanism. Furthermore, experiments in several species have shown that processes like the phase-advance mechanism are tuned to particular frequencies or rhythms dependent on the agents’ own signals to avoid interference from signals emitted by other species^21,22^. Hence, empirical data from these experiments is best explained by assuming a selective phase advance mechanism that favors signals with similar acoustic properties. Even though this mechanism is biased towards shortening periods, the single, shortened periods it produces cannot account for a continuous tempo increase.

In order to explain how the phase advance mechanism can lead to a continuous tempo increase, and thereby can result in joint rushing, a second component is needed. We assume that this role is played by a concurrently active period correction mechanism that may be unique to humans^23–26^. This mechanism picks up on the temporal differences coming from one-shot phase advancements and adjusts the internal timekeeper to the shorter interval. This leads to a shortening of all consequent intervals, i.e. a tempo increase. Possible candidates for period corrections of this kind are human-specific adaptation and anticipation mechanisms that govern interpersonal sensorimotor synchronization^10,27^. We propose that this combination of a simple synchronization mechanism observed in chorusing insects with a more sophisticated and human-specific mechanism could account for *joint rushing*. In order to test this proposal, we conducted three experiments using a simple synchronization-continuation task performed by small groups of participants.

### Experiment 1

In Experiment 1, we investigated whether rushing occurs specifically in joint performance, and if so, whether the presence of another (passive) person is enough to elicit rushing (an explanation based on social facilitation), or whether rushing is indeed contingent on the interaction with a co-actor. To do so, we asked participants each to strike a drum pad with a drumstick to perform a synchronization-continuation task in which a leading metronome fades away and participants are required to maintain the tempo either in an Individual or a Joint setting. Furthermore, we tested two potential factors that should foster joint rushing by increasing the chance of a signal falling into the sensitive window. First, we hypothesized that acting in larger groups (groups of three people) would elicit more rushing than acting in smaller groups (dyads) due to an increased chance of any co-actors’ signal falling into a partner’s sensitive window. Second, if a phase advance mechanism is involved in causing joint rushing, increasing each individual’s variability should also increase the chance of a signal falling into a partner’s sensitive window and should thereby result in a larger tempo increase. We used a manipulation of target force, i.e. participants were either instructed to strike the drum pad in a hard or soft manner. Lower force (striking the drum pad in a soft manner) should lead to higher variability^28^.

We calculated synchronization indices to assess the degree to which participants were able to follow the instructions of synchronizing with each other. A one-sample t-test showed that participants’ synchronization indices were significantly higher than a synchronization threshold of 0.73^29^ (*t*(23) = 7.685, *p* < 0.001, *d* = 1.569), with a mean of 0.86 and a standard deviation of 0.08. A further manipulation check showed that participants indeed struck the drum pads with a relatively low velocity in the low force condition (mean midi velocity = 25, SD = 3) and a high velocity in the high force condition (mean midi velocity = 113, SD = 12). A Welch t-test^30^ confirmed that this difference was statistically significant (*t*(23) = 41.092, *p* < 0.001, *d* = 10.384).

**Figure 2:**
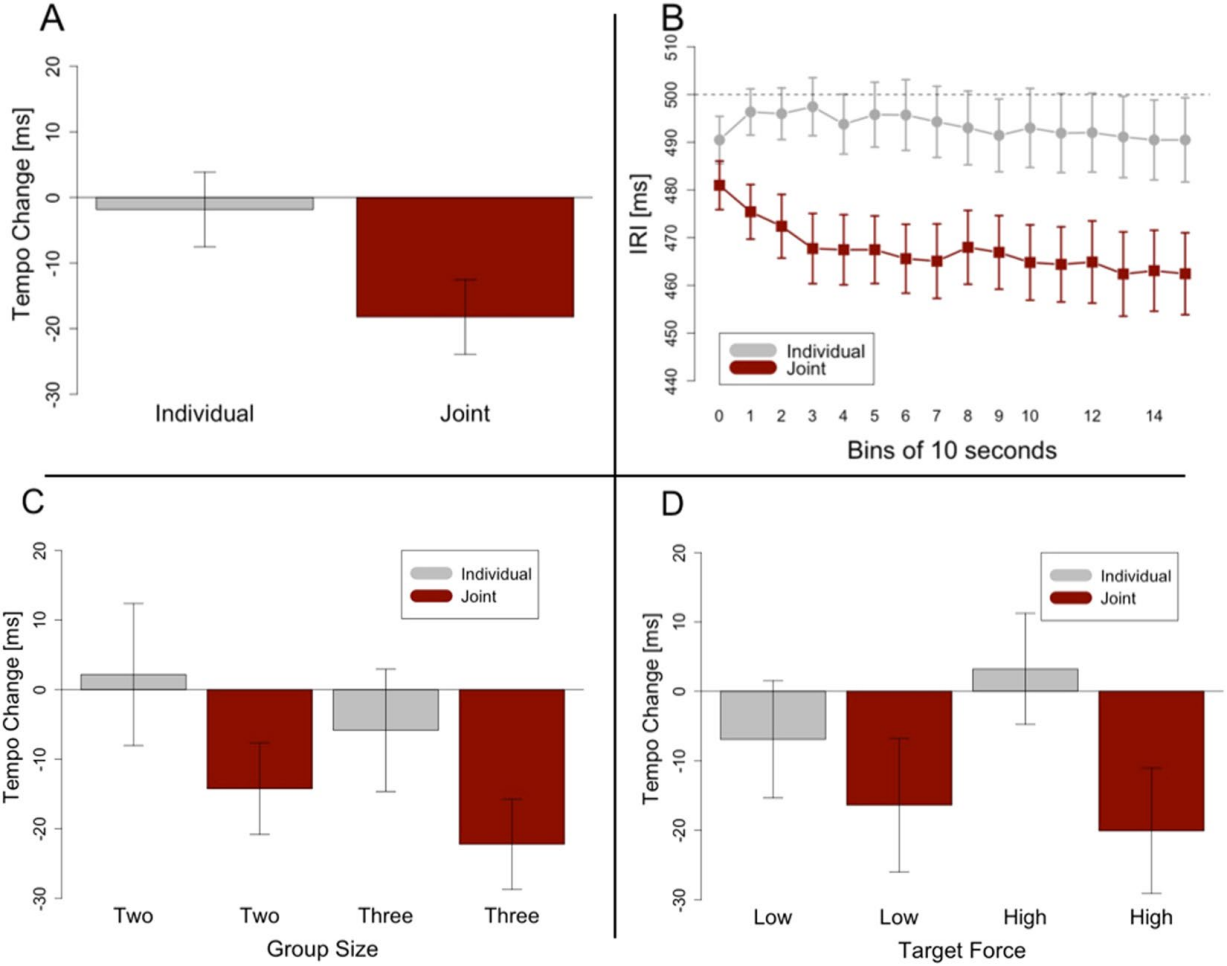
Results of Experiment 1. Tempo change indicates the difference between inter-response intervals at the end of a trial and at the beginning of a trial. Negative tempo change stands for a tempo increase. Error bars in (**A**,**D**) are calculated following the procedure recommended for within-subjects designs^42^. Error bars in (**B,C**) show standard errors. (**A**) Tempo change results for the individual and the joint condition. The higher negative tempo change in the joint condition is significantly different from zero. (**B**) The development of inter-response intervals over the course of trials, averaged over all participants and segmented into bins of 10 seconds each. (**C**) Effects of Task and Group Size on tempo change. Only the main effect for Task was significant. (**D**) Effects of Task and Target Force on tempo change. Only the main effect of Task was significant.

Tempo change was calculated in such a way that negative tempo change stands for shorter inter-response intervals (IRIs) at the end than at the beginning of a trial, which in turn stands for a tempo increase. In line with our prediction that the tempo should increase to a greater extent in joint performances, i.e. IRIs should become shorter, participants showed a more pronounced, negative tempo change in the joint condition (M = −18 ms, SD = 22 ms) than in the individual condition (M = −2 ms, SD = 35 ms), see Fig. 2A. A Welch t-test revealed that this difference was significant (*t*(23) = 3.170, *p* = 0.004, *d* = 0.59). One sample t-tests showed that the tempo increase in the individual condition was not significantly different from zero (*t*(23) = 0.277, *p* = 0.784), whereas the tempo change in the joint condition was different from zero (*t*(23) = 3.972, *p* < 0.001, *d* = 0.811). Thus, as expected, rushing occurred to a larger extent in the joint condition and was, in fact, absent during the individual condition. As there was always an experimenter present, we can conclude that the mere presence of another person was not sufficient to cause rushing. Figure 2B shows the data segmented into bins of 10 seconds to depict the average development of the tempo over the course of a trial split for joint and individual condition.

Group Size had no significant effect on tempo change (see Fig. 2C). A two-by-two mixed ANOVA with the within factor Task (Individual or Joint) and the between factor Group Size (Two or Three) revealed only a main effect for Task (*F*(1, 22) = 8.487, *p* = 0.008, *η*^2^ = 0.084), but no significant effect for Group Size (*F*(1, 22) = 0.630, *p* = 0.436), and no significant interaction (*F*(1, 22) < 0.001, *p* = 0.998). These results indicate that joint rushing occurred to the same extent in groups of two and groups of three.

In order to check whether higher target force led to higher temporal variability as intended by our manipulation, we calculated variability as squared residuals from a fitted linear model using time throughout each trial (in seconds) as a predictor of ITI, i.e. the tempo, to account for potential drift. We replicated the findings of a previous study^28^ in the individual condition. When participants were drumming individually, lower target force indeed resulted in higher values of residuals (M = 24.64, SD = 6.61) than higher target force (M = 20.69, SD = 5.21) (*t*(23) = 3.281, *p* = 0.003, *d* = 0.664). However, in the joint condition there was no significant difference in temporal variability between lower target force (M = 26.11, SD = 6.77) and higher target force (M = 26.11, SD = 11.02), (*t*(23) = 0.001, *p* = 0.999). In both joint conditions, variability was higher than in the individual condition with the higher variability. Therefore, there were no significant effects of the force manipulation on joint rushing. A 2 × 2 ANOVA with the within-subjects factors Task (Individual/Joint) and Target Force (high/low) showed a significant main effect of Task (*F*(1, 23) = 8.873, *p* = 0.007, *η*^2^ = 0.062), but no significant main effect of Target Force (*F*(1, 23) = 0.381, *p* = 0.543), and no significant interaction (*F*(1, 23) = 2.45, *p* = 0.131) (see Fig. 2D). The lack of significant effects of Force are most likely due to the fact that the force manipulation did not affect temporal variability as intended in the joint condition.

### Experiment 2

In Experiment 2, we tested whether producing same outcomes (i.e., the same pitched tones) leads to more rushing than when participants produce different outcomes (different pitched tones). This is predicted by a phase advance mechanism that is tuned to respond better, i.e. more strongly, to same outcomes than to different outcomes. In chorusing insects this phenomenon is believed to reduce cross-species interference^21,22^. Furthermore, we aimed to replicate the general finding of Experiment 1 that participants rushed more when they acted jointly with others than when they acted alone.

**Figure 3:**
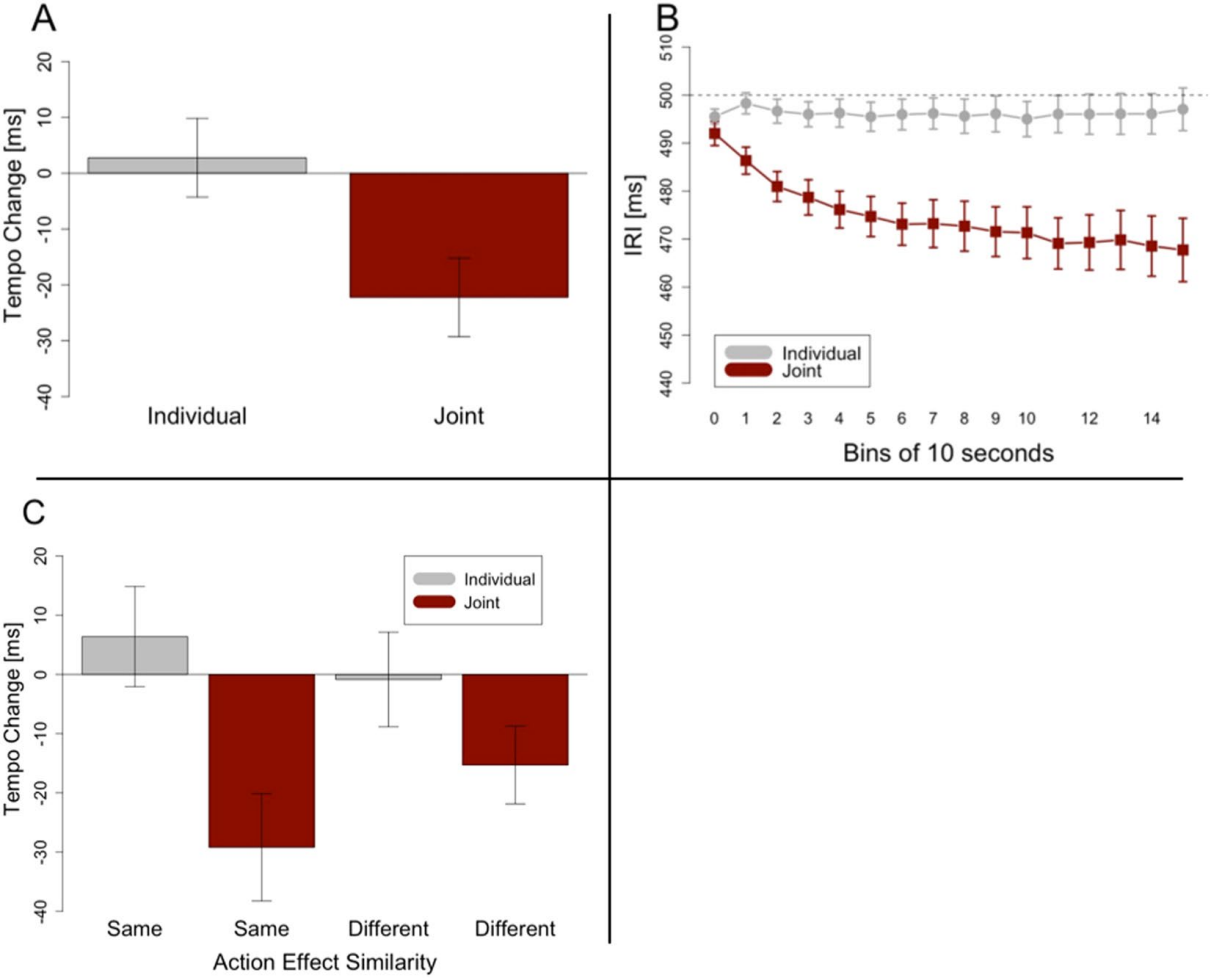
Results of Experiment 2. Tempo change indicates the difference between inter-response intervals at the end of a trial and at the beginning of a trial. Negative tempo change stands for a tempo increase. Error bars in (**A**,**C**) are calculated following the procedure recommended for within-subjects designs^42^. Error bars in B show standard errors. (**A**) This panel shows significantly more tempo change in the joint condition than in the individual condition. (**B**) The development of inter-response intervals over the course of trials, averaged over all participants and segmented into bins of 10 seconds each. (**C**) Effects of Task and Action Effect Similarity on tempo change. Post-hoc comparisons following a significant interaction revealed significantly more rushing in the joint condition with the same action effect than in the joint condition with a different action effect.

As in Experiment 1, a one-sample t-test showed that participants’ synchronization indices were significantly higher than the threshold of 0.73 (*t*(23) = 32.928, *p* < 0.001, *d* = 6.721) with a mean of 0.94 and a standard deviation of 0.03. As can be seen in Fig. 3A, participants showed a more negative tempo change in the joint condition (M = −22 ms, SD = 25 ms) than in the individual condition (M = 3 ms, SD = 19 ms), thereby replicating the overall joint rushing effect. A Welch t-test revealed that this difference was significant (*t*(23) = 3.673, *p* = 0.001, *d* = 1.125). Furthermore, tempo changes in joint trials were significantly different from zero (*t*(23) = 4.306, *p* < 0.001, *d* = 0.879), whereas tempo changes in the individual condition were not (*t*(23) = 0.727, *p* = 0.475). Figure 3B shows the data segmented into bins of 10 seconds to depict the average development of the tempo over the course of a trial.

A 2 × 2 ANOVA with the factors Task (Individual/Joint) and Action Effects (Same/Different) confirmed these results with a main effect of Task (*F*(1, 23) = 13.487, *p* = 0.001, *η*^2^ = 0.212) (see Fig. 3C). There was no significant main effect for Action Effects (*F*(1, 23) = 0.941, *p* = 0.342), but a significant interaction between Task and Action Effects (*F*(1, 23) = 15.278, *p* < 0.001, *η*^2^ = 0.046). Post-hoc t-tests showed that in the joint condition, there was a larger tempo increase for Same Action Effects (M = −29 ms, SD = 32 ms) than for Different Action Effects (M = 15 ms, SD = 21 ms) (*t*(23) = 3.421, *p* = 0.002, *d* = 0.511). The difference between those two in the individual condition was not significant (*t*(23) = 1.563, *p* = 0.132).

In Experiment 2, we tested whether producing the same sound versus producing a different sound than your partner has an effect on joint rushing. When the produced sounds were exactly the same joint rushing was more pronounced than when the sounds differed in pitch. This finding supports the assumptions that a selective phase advance mechanism is at work in joint rushing because it predicts that the higher similarity between own signal and other signal will increase the likelihood for corrections to occur.

### Experiment 3

In Experiment 3, we investigated whether performing the same actions—that is, movements with matching trajectories—has a similar effect on joint rushing as the similarity of action effects. This would be predicted by mirroring accounts^15^ that highlight facilitating effects of performing the same action. Such a facilitation could cause joint rushing. Alternatively, if a phase advance mechanism is involved in causing joint rushing, one would only expect this mechanism to be sensitive to signals that produce similar action effects (as demonstrated in Experiment 2) but not sensitive to another performing the same actions. The manipulation was implemented by asking participants to either strike the drum pads with the same movement trajectory, e.g. both strike a horizontally mounted drum pad with a vertical motion, or with different trajectories, i.e. one participant striking a drum pad with a vertical motion, while the other participant strikes a vertically mounted drum pad with a horizontal trajectory. Additionally, we aimed to replicate the general findings of Experiment 1 and 2 that participants rush more when they act jointly with others.

**Figure 4:**
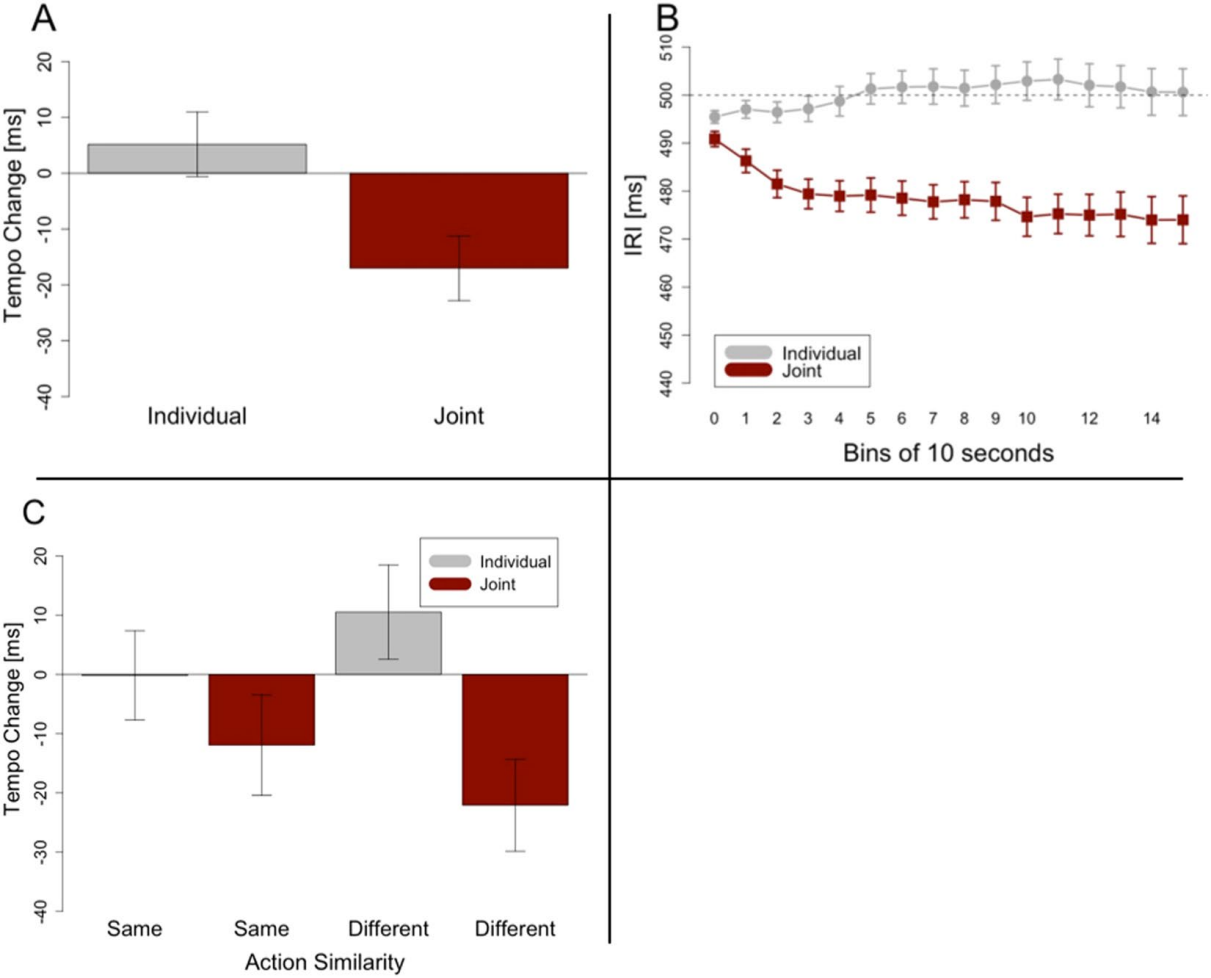
Results of Experiment 3. Tempo change indicates the difference between inter-response intervals at the end of a trial and at the beginning of a trial. Negative tempo change stands for a tempo increase. Error bars in (**A**,**C**) are calculated following the procedure recommended for within-subjects designs^42^. Error bars in B show standard errors. (**A**) This panel shows significantly more tempo change in the joint condition than in the individual condition. (**B**) The development of inter-response intervals over the course of trials, averaged over all participants and segmented into bins of 10 seconds each. (**C**) Effects of Task and Action Similarity on tempo change. Besides a significant main effect for Task, and a significant interaction, post-hoc comparisons revealed that the difference between the two Joint conditions (Same and Different) is not significant.

Synchronization indices were significantly higher than the threshold of 0.73 (*t(*23) = 71.339, *p* < 0.001, *d* = 14.562), with a mean of 0.94 and a standard deviation of 0.01. As in Experiments 1 and 2, we predicted the tempo change from trials in group settings to be negative and significantly different from/more negative than the tempo change from trials in solo settings. As shown in Fig. 4A, participants showed a more negative tempo change in the joint condition (M = −17 ms, SD = 21 ms) than in the individual condition (M = 5 ms, SD = 22 ms). A Welch t-test revealed that this difference was significant (*t*(23) = 3.964, *p* < 0.001, *d* = 1.042). As in experiments 1 and 2, the tempo change for group trials was significantly different from zero (*t*(23) = 4.041, *p* < 0.001, *d* = 0.825), whereas the tempo change for trials in which participants acted alone was not significantly different from zero (*t*(23) = 1.154, *p* = 0.260). Figure 4B shows the data segmented into bins of 10 seconds to depict the average development of the tempo over the course of a trial.

Furthermore, a 2 × 2 ANOVA with the factors Task (Individual/Joint) and Actions (Same/Different), revealed a main effect for Task (*F*(1, 23) = 15.713, *p* < 0.001, *η*^2^ = 0.166), but no main effect for Actions (*F*(1, 23) = 0.007, *p = *0.934) (see Fig. 4C). Surprisingly, the difference between Individual and Joint was larger in the Different Action condition than in the Same Action condition (*F*(1, 23) = 5.176, *p* = 0.033, *η*^2^ = 0.042). Post-hoc comparisons revealed that the only significant difference was between Individual Different and Joint Different (*t*(23) = 4.565, *p* < 0.001, *d* = 0.932).

The finding that performing similar actions does not enhance joint rushing demonstrates that joint rushing is unlikely to be a result of action mirroring. Rather, it indicates that joint rushing is caused by a phase advance mechanism that is tuned to specific auditory signals. An unexpected finding was that joint rushing was significantly larger when performing different actions than when performing same actions.

## General Discussion

Three experiments provided evidence that joint rhythmic performance leads to joint rushing. There was no evidence for rushing in the individual control conditions. The findings are in line with our hypothesis that rushing is due to the interaction with other people. We also conclude from our three experiments that joint rushing results from a phase advance mechanism that exhibits stronger effects when the two auditory signals share the same pitch. Such a phase advance mechanism is expected to introduce a bias towards interval shortening when different individuals produce auditory signals in a similar frequency range. Whereas in chorusing insects the shortening biases are local^11^, in humans additional anticipation and adaptation mechanisms are likely to pick up local biases and to transform them into adjustments of the period of an oscillating timekeeper, resulting in a global tempo change^31^.

We controlled for a number of alternative explanations for joint rushing. Social facilitation, speeding up because others are present in the same environment, cannot explain the joint rushing effects observed in the present study. In the current experiments, an experimenter was always present in the individual condition. According to arousal-based social facilitation, this should have led to some degree of rushing in the individual condition. However, across three experiments there was no indication of rushing in the individual condition. In the literature on music performance, it has been suggested that mental pressure could also lead to arousal and in turn to a tempo increase^32^. In a study by Yoshie, Kudo, Murakoshi and Ohtsuki^32^, an actual piano competition was held in a concert hall, including a large audience and five professional judges, to induce mental pressure in participating pianists. The findings show a tendency for faster tempo under conditions of mental pressure than during rehearsal. As neither an audience nor judges were part of our setup, the mental-pressure hypothesis should predict no rushing in the current study, neither in the individual nor in the joint condition. However, our findings across all three experiments consistently show that being engaged in coordination with another person leads to rushing. Hence, while we cannot generally rule out arousal as a factor inducing rushing, neither social facilitation^14^ nor an account based on mental pressure^32^ could explain the present results. It is also unlikely that action mirroring causes joint rushing. In Experiment 3 there was no support for the prediction derived from action mirroring theories that the extent of rushing should be higher when participants produce the same movements rather than different movements. Surprisingly, rushing effects were actually larger for different movements than for same movements. One potential explanation for this unexpected finding is that participants try consciously to counteract tempo changes that they notice (i.e., a contrast effect^33^); a process that may require cognitive control and exerting this control may be harder when different movements are performed.

The present findings are in line with two recent studies that have reported tempo increases during rhythmic activities in groups of different sizes. Okano, Shinya and Kudo^6^ analyzed the data of 24 adults required to tap their fingers in a synchronization-continuation paradigm, either in a paired or a solo condition, and found that rushing occurred to a larger extent in the joint condition. Okano *et al*. concluded that an interpersonal adaptation mechanism related to tap asynchrony underlies joint rushing. We add to this conclusion the proposal that a phase advance mechanism may be a central component of joint rushing.

Thomson, Murphy and Lukeman^7^ investigated synchronous clapping in groups of varying size. They found an asymmetrical period response curve with stronger corrections being made when a shortening of the period is required than when the period has to be prolonged. The proposed phase advance mechanism predicts stronger corrections for preceding signals than for following signals. Contrary to the results of Experiment 1, which showed no significant difference in joint rushing between groups of 2 and groups of 3, there was a positive correlation between group size and joint rushing in the Thomson *et al*. study, where group size varied from 7 to 220^7^. The proposed phase advance mechanism predicts such effects of group size because an increase in neighboring signalers translates into an increased chance of a signal falling into the sensitive window where a shortening of intervals occurs.

Given that robust effects of joint rushing have been established for different kinds of rhythmic performance at different tempi, the question arises how it is possible for humans to avoid joint rushing. According to our account, one way to reduce joint rushing would be to produce constantly low asynchronies between performers. This would prevent the occurrence of signals in the sensitive window, and thereby avoid interval shortenings. Thomson *et al*. reported incidental evidence that rhythmically trained individuals show a reduced effect of joint rushing^7^. Musical training is both known to lead to a reduction in motor/movement variability^34,35^ and to an improvement of tempo change detection abilities^36^. This would explain how (some) musical experts can accurately keep the tempo during joint music making.

While joint rushing can be a problem when the aim is a constant tempo during a joint music performance – according to the internet, the Rolling Stones and other rock bands seem to have struggled with this^1^ – joint rushing may have advantageous effects for other forms of joint action that put less constraints on tempo. People that have to coordinate their actions in time have been found to make themselves more predictable by reducing variability through an increase of their movement speed^37,38^. If more variability leads to more rushing, as Thomson *et al*. suggest^7^, and the increased tempo caused by joint rushing leads to a reduction in variability, then joint rushing could be part of a self-regulating mechanism. With this mechanism, the limiting factor for rushing would be the reduction in variability. Due to the nature of the sensitive window of the phase advance mechanism, phase advancement is not triggered anymore once the variability in the interaction falls below a certain threshold. Hence, joint rushing might ensure smooth interactions in groups by increasing tempo to the extent that this increase implies a reduction of variability.

Next steps in research on joint rushing could be to examine the influence of musical training on performance tempo in dyadic synchronization tasks, and to determine which components of musical expertise may allow interacting partners to reduce the effects of joint rushing. Furthermore, it would be valuable to investigate the relations among variability, asynchrony and joint rushing. Such an investigation could for example address the question whether joint rushing indeed functions as a coordination smoother^39^, i.e. whether it serves to simplify coordination in rhythmic joint actions. If joint rushing leads to smoother coordination, it could have provided a selective advantage in human evolution. This could explain why the phase advance mechanism, which we appear to share with other synchronizing species, is still a feature of human rhythmic behavior.

## Methods

For Experiment 1, we invited 24 participants (15 women, 8 men, 1 unspecified, mean age = 24.7 years, SD = 4.0 years), with little to no musical training (M = 0.4 years, SD = 1.3 years). The sample size was determined through a power calculation based on our expectation to obtain large effect sizes. This expectation was motivated by the fact that the joint rushing effect has been anecdotally reported to be perceivable without formal measurements. Post-hoc power analyses with the obtained effect sizes confirmed our intuitions, with power values ranging from 0.86 to 0.97 for the main comparisons across the three experiments. We kept the number of participants constant across all three experiments. Participants in all three experiments gave their informed consent and received gift vouchers as compensation. All experiments in this study were conducted in accordance with the Declaration of Helsinki and approved by the United Ethical Review Committee for Research in Psychology (EPKEB) in Hungary.

We used three Millenium MPS-400 Tom pads connected to a ddrum DDTi trigger interface to record responses, which participants produced with one wooden drum stick each. Auditory feedback, metronome beats, and data recording was handled with a custom Max MSP patch. Each participant produced a different piano pitch, with all pitches being more than an octave apart (15 semitones), centered around F#_4_.

Participants performed eight synchronization-continuation trials across four blocks. Each block consisted either of two trials where participants tapped alone (individual trials) or two trials of tapping in a group (group trials). The order of blocks was counterbalanced. During individual trials, a participant’s partner(s) waited in another room. In both individual and group trials, an experimenter was present and sat in close proximity to the participant(s) to control the program that presented stimuli and collected responses.

At the beginning of each trial participants heard a metronome with an Inter-Onset-Interval of 500 ms for 10 seconds. Participants were asked to synchronize with the metronome in the metronome phase and to then continue in the same tempo after the metronome faded out. After 150 seconds had elapsed and a minimum of 270 taps had been produced, participants heard a percussion sound informing them that the trial was over. After the experiment participants were asked to fill in a questionnaire from which we calculated the years of training on a musical instrument.

In Experiment 1, the main within manipulation was Task, which refers to whether participants performed the task alone (Individual) or in a group (Joint). Group Size was manipulated as a between factor, with half the participants performing the joint task in groups of two (Two) and half of the participants in groups of three (Three). Furthermore, we asked participants in 50% of the trials to strike the pads with a relatively low target force and in the remaining 50% of the trials with a relatively high target force. The low target force was introduced in an attempt to increase participants’ variability. Participants were provided with visual feedback that indicated to them whether they had struck the drum pads with the appropriate amount of force. The force of drum strokes was measured as MIDI velocity, which is coded in arbitrary units ranging from 1 (soft) to 127 (loud) that are proportional to the force of impact.

For Experiment 2, we invited 24 new participants (14 women, 10 men, mean age = 25.7 years, SD = 4.7 years), with little to no musical training (M = 0.0 years, SD = 0.1 years). The procedure was the same as in Experiment 1. Participants acted alone (Task: Individual) or in a group of two (Task: Joint). In Experiment 2 we also manipulated whether participants produced the same pitch (Same) or pitches that were 15 semitones apart (Different). This was implemented in such a way that for each participant it sounded as if the other’s pitch changed in the Same condition, i.e. for one participant both drum pads were heard as producing a D#_3_, whereas for the other participant it sounded as if both drum pads produced an F#_4_. This was made possible by using different channels for each participant’s auditory feedback. To sum up the design of Experiment 2, we used a 2 × 2 within-subjects design with the factors Task (Individual vs. Joint) and Action Effect (Same vs. Different).

For Experiment 3, we invited 24 new participants (16 women, 8 men, mean age = 25.0 years, SD = 3.8 years), with little to no musical training (M = 0.0 years, SD = 0.1 years). We kept the procedure the same as in Experiment 2 but exchanged the factor Action Effects with the new factor Action. For this experiment, we used two drum pads on each stand, one mounted horizontally and one mounted vertically. Instead of hearing the same or different pitches as in Experiment 2, participants struck the drum pad either in the same way (e.g. both hit the pad with a vertical movement) or in different ways (e.g. participant A hit the drum pad vertically, while participant B hit the drum pad horizontally). In both conditions, both drum pads produced a piano sound on C_4_. To summarize, in Experiment 3, we used a 2 × 2 within-subjects design with the factors Task (Individual vs. Joint) and Actions (Same vs. Different).

To determine how well participants followed the instruction to synchronize in the joint condition, we computed synchronization indices based on the circular variance of relative phase^38^. This unitless index reaches from 0, absence of synchronization, to 1, perfect synchrony. In line with the convention of previous studies, we considered indices > 0.73 to be indicative of the occurrence of synchronization^29,40,41^. To assess the tempo change over the course of a whole trial, we compute the difference between the mean of each participant’s inter-response intervals (IRIs) at the beginning, i.e. during the metronome phase, and for the mean of the last twenty IRIs for any given trial in milliseconds. Positive values of tempo change indicate that participants had larger IRIs at the end than at the beginning, i.e. slowed down, whereas negative values of tempo change indicate a shortening of IRIs, i.e. an increase in tempo.

## Data Availability

The datasets generated during and/or analyzed during the current study are available in the Open Science Framework repository, https://osf.io/j3dy5/?view_only=a3a70be72b8b415fb2afa863366f4c86.
